# Characteristics of patients associated with any outpatient antibiotic prescribing among Medicare Part D enrollees, 2007–2018

**DOI:** 10.1017/ash.2023.180

**Published:** 2023-06-29

**Authors:** Christine Y. Kim, Katryna A. Gouin, Lauri A. Hicks, Sarah Kabbani

**Affiliations:** 1 Epidemic Intelligence Service, Centers for Disease Control and Prevention, Atlanta, Georgia; 2 Division of Healthcare Quality Promotion, National Center for Emerging and Zoonotic Infectious Diseases, Centers for Disease Control and Prevention, Atlanta, Georgia

## Abstract

The 2007–2018 National Health Interview Survey data linked with Medicare claims were used to examine older adults’ characteristics and assess their associations with receiving an antibiotic prescription. This analysis shows variation in antibiotic prescribing among adults enrolled in Medicare Part D by race and ethnicity, sex, geography, and health status.

Antibiotic prescribing for older adults (aged ≥65 years) in outpatient settings accounts for a large proportion of antibiotic use, with a prescribing rate of 1,115 prescriptions per 1,000 persons in 2014.^1^ A better understanding of patient characteristics associated with antibiotic prescribing is important to identify potential opportunities to improve prescribing practices and reduce inequities in patient care. We examined antibiotic prescribing by self-reported sociodemographic and health characteristics among Medicare Fee-for-Service (those enrolled in Part A and Part B) and Part D beneficiaries during 2007–2013 and 2016–2018.

## Methods

Linked administrative claims data with data from the National Health Interview Survey (NHIS), a cross-sectional in-person national household survey of the US civilian, noninstitutionalized population, were used.^2^ The National Center for Health Statistics (NCHS) Research Ethics Review Board approved NHIS data collection and data linkage. The NCHS Data Linkage Program linked eligible NHIS respondents with the same individuals in the Centers for Medicare and Medicaid Services (CMS) Medicare enrollment and Part D prescription drug event (PDE) files during 2007–2018, based on the respondent providing consent and sufficient identifying information. Linkage-eligibility rates among respondents aged ≥65 years ranged from 48% to 88%, and match rates ranged from 90% to 96% for the study period.^3^ Part D PDE files were not linked in 2014–2015, so these years are excluded from the analysis.

The binary outcome variable was defined as any filled antibiotic prescription (vs none) in the PDE file during the respondent’s NHIS interview year. Predictor variables included demographic (ie, sex, age group, race and ethnicity, marital status, and education), health-related (ie, health status and multiple chronic conditions [MCCs]), geographic (ie, urbanicity and region), and socioeconomic characteristics (ie, imputed income-to-poverty ratio, ability to afford prescription drugs in the past 12 months, Medicare and Medicaid dual eligibility, and low-income subsidy status), and survey year. All variables were self-reported except for dual eligibility, low-income subsidy status, and MCC; all were obtained from the CMS data. MCC included 15 conditions consistent with the Health and Human Services MCC list using an approach applied in the published literature.^4^

**Figure 1.**
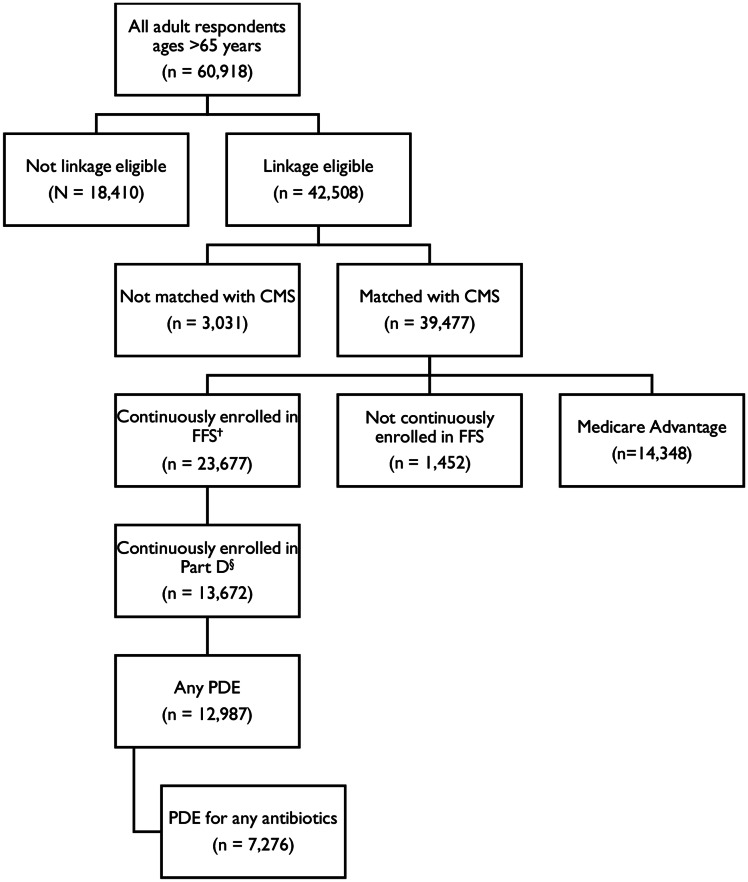
Flowchart of analytic sample among NHIS respondents with Medicare enrollment, 2007–2013 and 2016–2018. Note. CMS, Centers for Medicare and Medicaid Services; FFS, fee for service; PDE, prescription drug event. *Excludes NHIS data from 2014–2015 because these were not linked with PDE data files. ^†^NHIS respondents aged >65 years and were enrolled continuously for 12 months in the Master Beneficiary Summary File for Part A and Part B during the calendar year of their survey interview. ^§^NHIS respondents aged >65 years and were enrolled continuously for 12 months in Part D during the calendar year of their survey interview. SOURCE: National Center for Health Statistics, National Health Interview Survey, 2007–2013 and 2016–2018, linked to Centers for Medicaid and Medicare Services, 2007–2013 and 2016–2018.

Analyses were limited to linked survey respondents aged >65 years at the time of their interview with complete data across all model variables and continuous enrollment for Part A, Part B, and Part D during the survey calendar year who had at least 1 PDE claim, so their full-year drug use could be observed. We excluded those without any claims because the Part D data do not differentiate between whether the beneficiary never received a prescription, chose not to fill, or filled their prescriptions in ways that were not captured in the data.^5^ During 2007–2013 and 2016–2018, 60,918 adults aged >65 years were interviewed, 39,477 were matched with CMS data, 13,672 adults were continuously enrolled in Part D and Medicare fee-for-service, and 12,987 had any PDE claims (Fig. 1). All analyses accounted for the survey’s multistage, complex sampling design and used linkage-eligible adjusted weights. Survey sample weights were adjusted for linkage eligibility (nonresponse).^2^

We examined patients’ characteristics and assessed their associations with receiving an antibiotic prescription using a logistic regression model. We tested interaction terms between ability to afford prescription medications and race and ethnicity and ability to afford prescription medications and multiple chronic conditions because antibiotic prescribing may vary by these variables; these results were statistically insignificant and were not included.^6,7^ Due to multicollinearity, imputed income-to-poverty ratio and low-income subsidy variables were removed from the model. Analyses were conducted using SAS version 9.4 software (SAS Institute Inc, Cary, NC) and SAS-callable SUDAAN version 11.0 software (RTI International, Research Triangle Park, NC).

## Results

Individual characteristics and adjusted odds ratios are shown in the Table 1. Part D beneficiaries with any PDE claims were overall more likely to live in urban areas, to be female, to be non-Hispanic White, to have self-reported excellent or very good/good health status, and to have ≤3 chronic conditions. Characteristics associated with any antibiotic prescribing included female versus male sex (1.31; 95% CI, 1.21–1.48), being married versus not married (1.18; 95% CI, 1.07–1.31), with fair or poor health status vs. excellent or very good/good (1.19; 95% CI, 1.05–1.34), >1 chronic condition versus 0 or 1 (2–3 conditions, 1.32; 95% CI, 1.16–1.49; 4–5 conditions, 1.91; 95% CI, 1.58–2.07; ≥6 conditions, 3.13; 95% CI, 2.62–3.74), reside in the Southern region versus the Northeast (1.18; 95% CI, 1.03–1.36), and unable to afford prescription drugs (1.41; 95% CI, 1.11–1.79). Non-Hispanic Black adults (0.61; 95% CI, 0.51–0.87) and non-Hispanic Asian adults (0.65; 95% CI, 0.49–0.87) were less likely to receive an antibiotic prescription compared to non-Hispanic White adults.

**Table 1.** Selected Characteristics of US Adults (aged >65 years) with Medicare Part D and Associations with Antibiotic Prescribing, United States, 2007–2013 and 2016–2018

Characteristic	Part D (n = 13,672)		Any PDE^a^ (n = 12,062)^c^
	No.	% (95% CI)^b^	aOR (95% CI)
**Demographic characteristics**			
Sex			
Male	5,063	38.6 (37.5–39.6)	Ref
Female	8,609	61.4 (60.4–62.5)	1.31 (1.21–1.48)
Age group			
66–69 y	3,270	24.9 (24.0–25.8)	Ref
70–74 y	3,703	28.0 (27.0–28.9)	1.03 (0.91–1.18)
75–79 y	2,744	19.7 (18.8–20.6)	1.02 (0.88–1.18)
80+ y	3,955	27.4 (26.4–28.4)	0.88 (0.77–1.01)
Race/ethnicity			
Hispanic	738	5.4 (4.7–6.1)	0.96 (0.76–1.21)
Non-Hispanic Asian	422	3.4 (3.0–4.0)	0.65 (0.49–0.87)
Non-Hispanic Black	1,189	6.2 (5.7–6.9)	0.61 (0.51–0.87)
Non-Hispanic White	11,212	84.2 (83.1–85.2)	Ref
Non-Hispanic Other	111	0.7 (0.5–1.0)	0.78 (0.44–1.37)
Marital status			
Married/living with partner	5,578	54.1 (52.9–55.4)	1.18 (1.07–1.31)
Not married/not living with partner^d^	8,085	45.9 (44.6–47.1)	Ref
Education			
<High school	2,933	20.8 (19.8–21.8)	Ref
High school graduate/GED	4,021	29.9 (28.9–30.9)	1.06 (0.92–1.21)
Some college/No degree	2,218	16.2 (15.4–17.0)	1.00 (0.85–1.18)
College graduate	4,449	33.1 (32.0–34.3)	1.16 (1.00–1.35)
**Health-related characteristics**			
Health status			
Excellent/very good/good	10,318	75.4 (74.4–76.3)	Ref
Fair/poor	3,353	24.6 (23.7–25.6)	1.19 (1.05–1.34)
Multiple chronic conditions^e^			
0–1 conditions	3,490	25.8 (24.8–26.7)	Ref
2–3 conditions	4,834	36.1 (35.0–37.1)	1.32 (1.16–1.49)
4–5 conditions	3,314	24.3 (23.5–25.2)	1.81 (1.58–2.07)
≥6 conditions	1,945	13.8 (13.1–14.7)	3.13 (2.62–3.74)
**Geographic characteristics**			
Urbanicity			
Urban	9,423	74.4 (72.8–75.8)	Ref
Rural	3,465	25.6 (24.2–27.2)	0.99 (0.89–1.12)
Region			
Northeast	2,297	18.0 (16.8–19.3)	Ref
Midwest	3,619	26.4 (25.0–27.8)	1.08 (0.94–1.24)
South	5,043	36.7 (35.2–38.4)	1.18 (1.03–1.36)
West	2,613	18.9 (17.5–20.3)	1.05 (0.91–1.22)
**Socio-economic characteristics**			
Income-to-Poverty Ratio			
<100%	2,055	12.0 (11.2–12.9)	…
100%–199%	3,462	23.6 (22.5–24.7)	…
200%–399%	4,231	32.0 (30.9–33.1)	…
>400%	3,924	32.4 (31.1–33.6)	…
Could not afford prescription, past 12 mo			
No	12,990	95.5 (95.0–95.9)	Ref
Yes	621	4.5 (4.1–5.0)	1.41 (1.11–1.79)
Dual eligibility			
No	11,034	83.3 (82.2–84.3)	Ref
Yes	2,638	16.7 (15.7–17.8)	1.10 (0.95–1.27)
Low-income subsidy			
100% premium, no copay	1,836	12.2 (11.3–13.2)	…
25–100% premium, med–high copay	1,466	9.1 (8.5–9.8)	…
No subsidy	10,370	78.6 (77.4–79.8)	…

Note. aOR, adjusted odds ratio; PDE, prescription drug event; Ref, referent category.

All variables are from NHIS except for dual eligibility, low-income subsidy, multiple chronic conditions, and outcomes, which are from the Medicare claims data.

Interaction terms were not statistically significant, so they were not included in the model (affordability and multiple chronic conditions, *P* = .058, affordability and race and ethnicity, *P* = .746).

Variables included in the model include those with corresponding aORs. Models also adjusted for survey year. SOURCE: National Center for Health Statistics, National Health Interview Survey, 2007–2013 and 2016–2018 linked to Centers for Medicaid and Medicare Services, 2007–2013 and 2016–2018.

a PDE for antibiotics versus none.

b Weighted estimates and adjusted for complex survey design.

c Complete case analysis was used for the logistic regression model, so sample size does not match the figure in the supplement.

d Includes separated, divorced, widowed, and never married.

e These included hypertension, congestive heart failure, coronary artery disease, cardiac arrhythmias, hyperlipidemia, stroke, arthritis, asthma, cancer (female breast cancer, colorectal cancer, prostate cancer, lung cancer), chronic kidney disease, chronic obstructive pulmonary disease, dementia, depression, diabetes, and osteoporosis.

## Discussion

We used a unique data set with self-reported demographic and socioeconomic characteristics linked to claims data to characterize antibiotic prescribing among US older adults with Medicare Part D. Although the population had continuous healthcare coverage, this analysis shows variation in antibiotic prescribing among older adults by race and ethnicity, sex, geography, and health status. Prior research has shown that Black children were less likely to receive antibiotics than White children,^6^ and similar findings have been described among older adults inappropriately prescribed an antibiotic for COVID-19.^8^ This analysis shows that non-Hispanic Black older adults were less likely to receive antibiotics, which may represent a health inequity warranting further study. Women and people with MCCs were more likely to be prescribed antibiotics, which may reflect more need for antibiotics, healthcare-seeking behavior, or healthcare exposure. Higher antibiotic prescribing rates in the South have also been described in the literature and these geographic variations persisted after adjusting for other characteristics.^9^ The inability to afford prescription drugs was also associated with more prescribing, which may reflect delayed care or potential higher severity of illness.

Limitations include a small sample size, linkage eligibility, and pooled cross-sectional data limiting the evaluation of cumulative antibiotic prescribing, prescribing appropriateness, or trends over time. However, we used self-reported sociodemographic data often unavailable in other commonly used data sources. Although CMS administrative data include imputed race and ethnicity, analyses are often restricted to White and Black persons because of the low validity of other race and ethnicity categories.^10^ We assumed that survey responses applied to the entire period, regardless of when the interview occurred; this may not reflect an individual’s situation, particularly if interviewed earlier in the calendar year. We included respondents with any claim but did not capture those who may have received a prescription that was not filled or was filled in ways not captured in the data. Only 5% of those fully enrolled in Part D did not have a claim. We did not analyze condition-specific prescribing by outpatient visits to observe overall associations of prescribing in the older adult population, limiting comparisons with other studies on condition-specific prescribing. Medicare claims data do not capture free and low-cost medications,^5^ which may attenuate the effect of self-reported inability to afford prescription drugs. Our model used a control for dual eligibility, which may account for some confounding by socioeconomic status. Finally, our study population was limited to the civilian noninstitutionalized population with Part D coverage and may not be representative of all Medicare beneficiaries.

This analysis shows variation in antibiotic prescribing by patient characteristics in older adults with continuous access to health insurance and prescription drug coverage during the study period. Future analyses may consider the evaluation of prescribing appropriateness, and this data set could be used to explore complex health-equity questions for common health conditions in older adults. Efforts to improve antibiotic prescribing quality for older adults should incorporate a health-equity lens to ameliorate rather than exacerbate disparities.
